# Free Artificial Teeth for Soldiers

**Published:** 1894-12

**Authors:** 


### Editorial, Etc.

FREE ARTIFICIAL TEETH FOR SOLDIERS.—After careful consideration of the question, the Emperor of Germany and the Berlin War Office have now decided that artificial teeth are to be provided free of charge to the soldiers, on the ground that the troops are better able to render good service with sound grinders than with teeth which either ache or are inadequate to the performance of their work of mastication.

